# A High-Accuracy Solid/Liquid Composite Packaging Method for Implantable Pressure Sensors

**DOI:** 10.3390/mi17020162

**Published:** 2026-01-27

**Authors:** Bo Wang, Yubiao Zhang, Yuning Huang, Zhonghua Li, Senran Jiang, Fuji Wang, Qiang Liu, Xing Yang

**Affiliations:** 1 School of Mechanical Engineering, Dalian University of Technology, Dalian 116024, China; wangbo12@mail.dlut.edu.cn; 2 Department of Precision Instrument, Tsinghua University, Beijing 100084, China; zhangyubiao0206@163.com (Y.Z.); 2023200013@buct.edu.cn (Y.H.);; 3 State Key Laboratory of Precision Space-Time Information Sensing Technology, Beijing 100084, China; 4 Key Laboratory of Photonic Control Technology, Tsinghua University, Ministry of Education, Beijing 100084, China

**Keywords:** MEMS, pressure sensor, Parylene-C, silicone oil, composite packaging structure

## Abstract

This study addresses the critical packaging requirements of implantable pressure sensors concerning measurement accuracy and environmental stability. We propose a solid/liquid composite packaging technique based on Parylene-C and silicone oil. Utilizing liquid silicone oil as an intermediate medium, this method effectively decouples solid/solid interface shear forces, thereby mitigating measurement errors caused by mechanical coupling. Furthermore, the superior hydrophobic properties of silicone oil and its defect-filling capability are employed to slow the infiltration rate of water molecules at the interface, ensuring long-term stability. The influence of the solid/liquid composite layer on the mechanical properties of the sensor’s sensitive element was analyzed through finite element simulation. The experimental results demonstrate the efficacy of this approach: after adding a liquid silicone oil layer between the Parylene coating and the sensitive element, the sensor’s accuracy improved to 0.5 mmHg within the pressure range encountered in clinical human applications. In simulated bodily fluids, it demonstrated exceptional long-term stability, with drift values consistently below 2 mmHg over a 30-day period. This research provides a feasible and straightforward solution for the packaging design of high-performance implantable pressure sensors.

## 1. Introduction

With advances in Micro-Electro-Mechanical Systems (MEMS) technology, implantable pressure sensors have become core components in real-time physiological monitoring and intelligent diagnostic systems. In complex physiological environments, such as the eyeball [1,2], cranial cavity [3,4], and heart [5,6], these sensors must simultaneously meet stringent requirements for high-precision measurement, long-term stable operation, and excellent biocompatibility, which poses significant challenges to existing packaging technologies [7]. Among various solutions, the polymer Parylene-C has emerged as a research hotspot [8,9]. Utilizing the chemical vapor deposition (CVD) process, Parylene-C forms a highly conformal, void-free thin film that provides effective environmental protection while maintaining a compact device size [10]. Parylene-C inherently boasts low moisture permeability, excellent corrosion resistance, and superior biocompatibility [11,12]. Specifically, the film prevents internal circuits from being encroached upon by water vapor in bodily fluid environments, guaranteeing stable operation in vivo [13,14].

Despite these advantages, the durability of Parylene-C coatings for long-term waterproofing remains limited, primarily due to a dual problem [15,16]: First, as a polymer, its molecular chain density results in a degree of water permeability, where liquid gradually reaches the device surface through permeation, leading to long-term failure [17,18,19]. Second, significant shear stress is generated at the interface between the coating and the sensor’s sensitive element due to repeated pressure-induced deformation [20]. This shear stress not only causes direct measurement errors but also triggers interface delamination and coating degradation/cracking upon water infiltration, accelerating liquid penetration and forming a vicious cycle, and both accuracy and sensitivity will be significantly adversely affected as a result. The existence of this dual problem severely restricts the application of Parylene-C coatings in high-precision, long-term implantable pressure sensors [21,22].

H. Xu et al. proposed improving adhesion by forming a Parylene-C/F copolymer coating through co-deposition of Parylene-C and fluorine powders. Compared with pure Parylene-C or Parylene-F coatings, the copolymer layer exhibited substantially higher bonding strength [23]. Sun et al. introduced a Parylene-C/PDMS flexible encapsulation method that preserves sensor accuracy (0.2 mmHg) and sensitivity (99.72%). With machine learning-based blood pressure estimation, systolic and diastolic accuracy improved by 31.4% and 21% over traditional methods [24]. Buchwalder et al. enhanced the Parylene-encapsulated sensor by applying an additional hydrophilic SiO_2_ coating. This surface modification effectively prevented encrustation after bladder implantation, making the sensor well suited for long-term urological use [25]. Current research mainly focuses on enhancing the adhesion between the encapsulation layer and the sensor’s sensitive element to counter interfacial slip, delamination failure caused by significant shear forces, and the resulting decrease in measurement accuracy and device failure due to water infiltration. However, this approach still struggles to consistently withstand the long-term fatigue induced by this intrinsic mechanical coupling stress.

To address this challenge, this study proposes a novel solid/liquid composite packaging structure composed of a liquid silicone oil intermediate layer and a solid Parylene-C thin film. The silicone oil, acting as a fluid with zero shear stress, effectively isolates the mechanical coupling stress between the outer encapsulation layer and the sensor’s sensitive element, fundamentally reducing measurement errors. Concurrently, the hydrophobic nature of the silicone oil further enhances waterproofing capabilities, thereby extending the device’s lifespan. The Parylene-C film, deposited on the surface of the silicone oil, ensures the overall structure’s biocompatibility and environmental isolation. We utilize Finite Element Method (FEM) simulation to analyze the impact of this solid/liquid composite packaging layer on the mechanical properties of the sensor’s sensitive element [26]. This research aims to achieve structural optimization, offering a new solution for the packaging of implantable pressure sensors that are highly precise, long-term stable, and suitable for in vivo applications.

## 2. Experimental Setup

### 2.1. Packaging Preparation

To systematically analyze the influence of the proposed solid/liquid composite encapsulation method on sensor performance, a comprehensive set of comparative experiments was designed. Three distinct groups of sensors were fabricated for testing: unpackaged, bare sensor chips, which served as the performance benchmark; single-layer Parylene encapsulation; and sensors employing the novel Parylene/silicone oil solid/liquid composite packaging method proposed in this study. Furthermore, to specifically investigate the effect of Parylene-C thickness on sensor performance, both the single Parylene-C encapsulation group and the solid/liquid composite encapsulation group were fabricated with varied thicknesses of the Parylene-C layer for a detailed comparative study.

All the reagents and solvents are common and readily available materials that can be used directly without further purification. This experiment takes the 5420C sensor from the SMI company (Milpitas, CA, USA) as an example. The solid/liquid composite encapsulation in this study primarily involves two steps: the spin coating of the silicone oil protective layer and the deposition of the Parylene-C thin film. During pre-encapsulation pretreatment, first use a nitrogen gun to blow away large dust particles from the chip surface. Then wipe the sensor surface with cotton swabs dipped in anhydrous ethanol. Finally, blow-dry with nitrogen and set aside for later use.

Silicone Oil Layer Formation: The protective silicone oil layer was prepared on the surface of the MEMS pressure sensor’s sensitive element using a spin coating technique. Initially, the pretreated sensor chip was secured onto the center of the spin coater stage. Approximately 2 mL of degassed silicone oil was then precisely dispensed onto the central area of the sensitive element using a precision dispensing system. The spin coating process followed a two-stage program: First, the substrate was rotated at 800 rpm for 15 s to allow the silicone oil to fully spread across the entire sensitive region via centrifugal force. Subsequently, the rotation speed was rapidly increased to 6000 rpm and maintained for 60 s. The strong shear force generated by the high-speed rotation effectively removed excess silicone oil, resulting in a uniform and stable silicone oil thin film.

Parylene-C Vapor Deposition [27,28]: Following the silicone oil application, a Parylene-C film coating was formed on the chips using a specialized coating system (Model PDS2010, Specialty Coating Systems, Inc., Indianapolis, IN, USA). The sensor chips were loaded into the deposition chamber, and the corresponding mass of solid Parylene-C dimer precursor was placed into the vaporization boat. The system was then evacuated until the deposition chamber reached a vacuum of 10 mTorr. Next, the precursor in the vaporization boat was heated to 175 °C for sublimation. The resulting gaseous dimer entered the pyrolysis furnace, where it was cracked into gaseous monomers at a high temperature of 690 °C. These gaseous monomers finally entered the vacuum deposition chamber, where they deposited and polymerized on the silicone oil surface of the pressure sensor chips, forming the required Parylene-C protective thin film.

### 2.2. Packaging Structure

**Figure 1:**
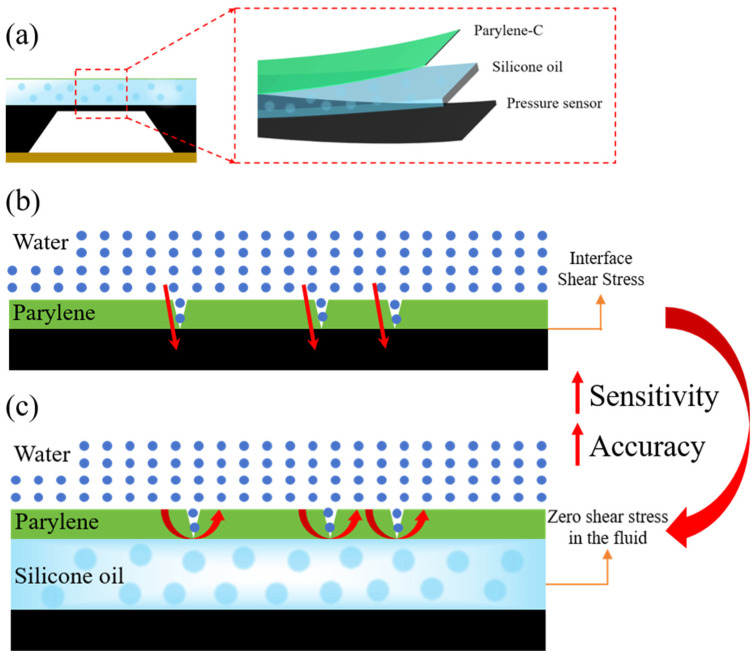
(**a**) Solid/liquid composite packaging structure; (**b**) schematic diagram showing water molecule permeation in the Parylene packaging structure; (**c**) schematic diagram showing water molecule permeation in the solid/liquid composite packaging structure.

Figure 1a illustrates a schematic of the solid/liquid composite encapsulation structure. The pressure sensor, serving as the core sensitive element, is located at the bottom. Its sensitive diaphragm is closely covered by a silicone oil layer (blue) prepared via spin coating. This liquid medium primarily functions to eliminate shear forces and enhance anti-permeation performance. Figure 1b illustrates the water molecule permeation process and the resulting failure under a single-layer Parylene encapsulation. Intrinsic micro-pores or defects within the Parylene coating provide permeation pathways for water molecules. More critically, under prolonged operation or mechanical fatigue from external loads, significant interfacial shear stress develops between the coating and the underlying sensor surface. This shear force drives delamination and microcrack formation within the coating. The resulting delamination and cracks further accelerate water molecule permeation rates, ultimately leading to sensor failure due to water ingress. In contrast, Figure 1c illustrates a solid/liquid composite encapsulation structure incorporating a silicone oil layer and its anti-permeation mechanism. The silicone oil layer, acting as a hydrophobic fluid medium, provides a primary physical barrier against water molecules. More critically, it isolates the rigid contact between the upper Parylene coating and the underlying sensor surface, achieving the ideal state of zero shear stress in the fluid. By eliminating or substantially reducing the interfacial shear stress responsible for delamination and cracking, this composite encapsulation structure significantly enhances the sensor’s long-term reliability and impermeability.

**Figure 2:**
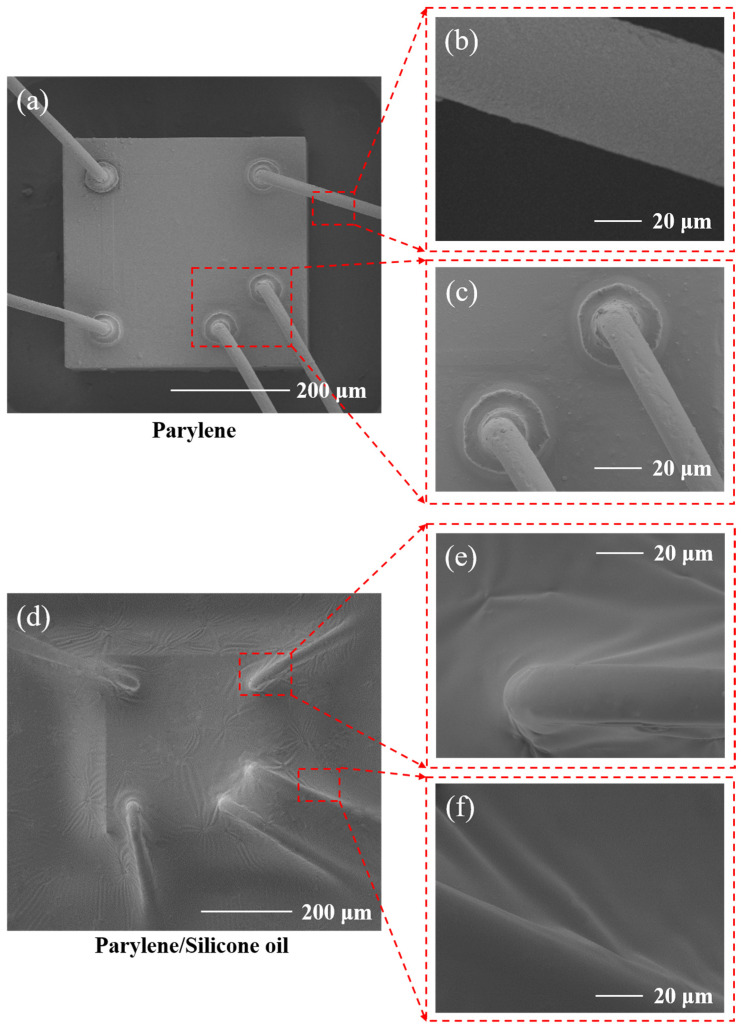
(**a**) SEM photo of the Parylene packaged sensor; (**b**,**c**) SEM photos of the Parylene packaging near the gold wires; (**d**) SEM photo of the Parylene/silicone oil composite packaged sensor; (**e**,**f**) SEM photos of the composite packaging near the gold wires.

Figure 2 presents the scanning electron microscope (SEM) images of the sensor after being encapsulated with Parylene and Parylene/silicone oil. The thickness of the Parylene film is 1 μm. As shown in Figure 2a–c, the Parylene deposited on the sensor surface exhibits conformal properties, clearly revealing the contours of the sensor. In Figure 2d–f, the silicone oil layer can be clearly observed as a stable underlying support near the gold wire bonding area, effectively supporting the ultra-thin Parylene film. No obvious structural defects, pinholes, or delamination issues were found on the encapsulation surface, which collectively confirms that the dense and conformal solid/liquid composite encapsulation layer has been successfully and high-quality fabricated. This further indicates that this composite structure provides a gas-tight and mechanically stable protection for the sensing chip.

### 2.3. Testing Methodologies and Platform

The pressure measurement range for this study was set from 104 kPa to 114 kPa (780.1 mmHg to 855.1 mmHg), with a pressure increment of 2 kPa (15.0 mmHg). This range covers implantable physiological monitoring near atmospheric pressure, such as intravesical pressure (104–110 kPa), intraocular pressure (~105 kPa), and arterial blood pressure (~112 kPa). The sensor output corresponding to the input pressure points was recorded, and the ascending and descending pressure calibration cycles were repeated three or more times. The sensor error calculation formula is as follows [29].

Sensitivity:

(1) $$k=\frac{∆y}{∆x}\times 100\%$$

where ∆y is the output voltage increment, and ∆x is the input voltage increment.

Nonlinear error:

(2) $$\xi_{L}=\frac{{\left|{\bar{y}}_{i}-y_{i}\right|}_{max}}{Y_{FS}}\times 100\%$$

where ${\bar{y}}_{i}$ is the mean output of the *i*-th set, and the full-scale output value is $Y_{FS}$.

Hysteresis error:

(3) $$\xi_{H}=\frac{{\left|∆{\bar{y}}_{i}\right|}_{max}}{Y_{FS}}\times 100\%$$

where $∆{\bar{y}}_{i}$ refers to the average difference in forward and reverse outputs for the *i*-th set.

Repeatability error:

(4) $$\xi_{R}=\frac{3S}{Y_{FS}}\times 100\%$$

where *S* refers to the sample standard deviation of the sensor outputs over the entire measurement range.

Accuracy (Total Error) [29]:

(5) $$\xi =\sqrt{{\xi_{L}}^{2}+{\xi_{H}}^{2}+{\xi_{R}}^{2}}$$

**Figure 3:**
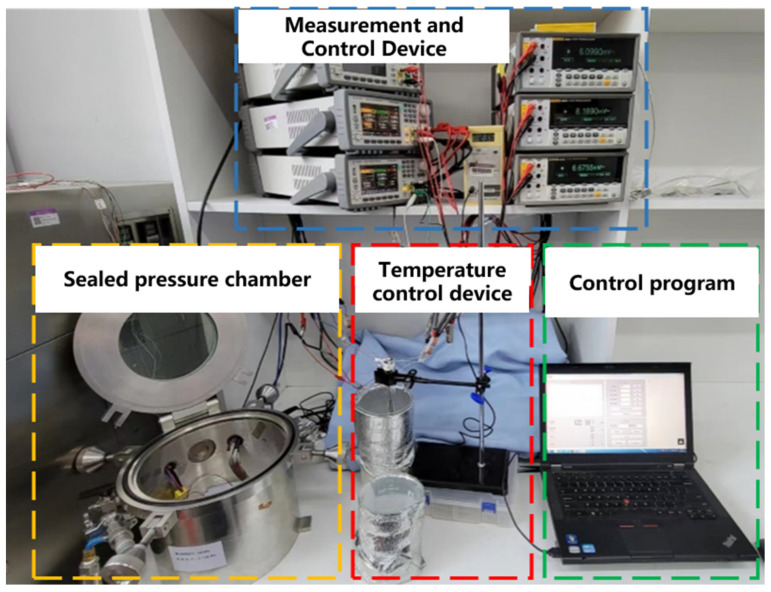
Pressure sensor testing platform.

A dedicated pressure sensor testing system was designed in this study to evaluate the accuracy and stability of the fabricated pressure sensors. This system boasts high-precision pressure control capabilities, with a control accuracy reaching ±1 Pa (0.0075 mmHg). The structure is shown in Figure 3. The pressure sensor measurement system primarily consists of a pressure measurement and control subsystem, an electrical parameter testing subsystem, a temperature control subsystem, and a sealed pressure chamber. It enables data measurement of implantable pressure sensors at specified pressures. The system regulates gas flow by adjusting the pressure micro-pump within the pressure measurement and control subsystem and the opening degree of the pneumatic valve in the sealed pressure chamber. It determines the sensor’s precision metrics by measuring the output voltage of the implantable pressure sensor at different pressure values during periodic step changes. This measurement is performed using the high-precision pressure gauge in the pressure measurement and control subsystem and the instruments in the electrical parameter testing subsystem. Furthermore, the system integrates temperature control functionality, which is utilized for conducting anti-body fluid corrosion (immersion) lifetime tests on the encapsulated pressure sensors to assess their long-term stability.

## 3. Results and Discussion

### 3.1. Finite Element Analysis (FEA)

In the packaging of piezoresistive pressure sensors, the complex shear stress generated at the solid/solid interface between the Parylene coating and the sensor’s sensitive element is a critical factor leading to zero-point drift, hysteresis, and even interfacial delamination failure of the sensor. This issue severely restricts the application of Parylene-C coatings in high-precision, long-term implantable pressure sensors.

This study employed the finite element simulation method to conduct a simulation analysis on the implantable MEMS pressure sensor based on the deformable diaphragm structure, aiming to evaluate the influence of the proposed solid/liquid composite packaging structure on the mechanical behavior of the sensor’s sensitive unit. In the simulation, the sensor chip was simplified as an inverted square silicon cup structure, and it was assumed that the sensor was subjected to a uniformly distributed pressure load, with the equivalent pressure load set at 0.12 MPa.

**Figure 4:**
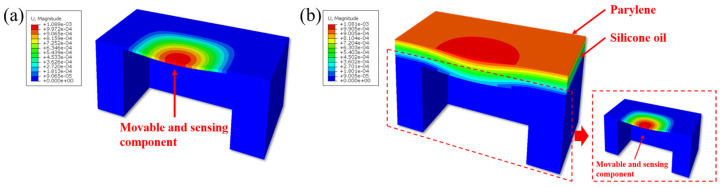
(**a**) Deformation of the bare sensor; (**b**) deformation of the Parylene/silicone oil composite sensor.

Figure 4a,b illustrate the visualization results of the deformation of the sensor’s sensitive element before and after solid/liquid composite encapsulation. It can be observed that the deformation magnitude of the sensor’s sensitive element remains nearly unchanged, decreasing by only 0.64%. In both cases, the maximum deformation is consistently located at the center of the sensitive element. This result demonstrates that the fundamental mechanical response of the sensor—the “sensing mechanism” of converting external pressure signals into self-deformation—is preserved. This preservation inherently ensures the accuracy and reliability of the measurement results after encapsulation.

**Figure 5:**
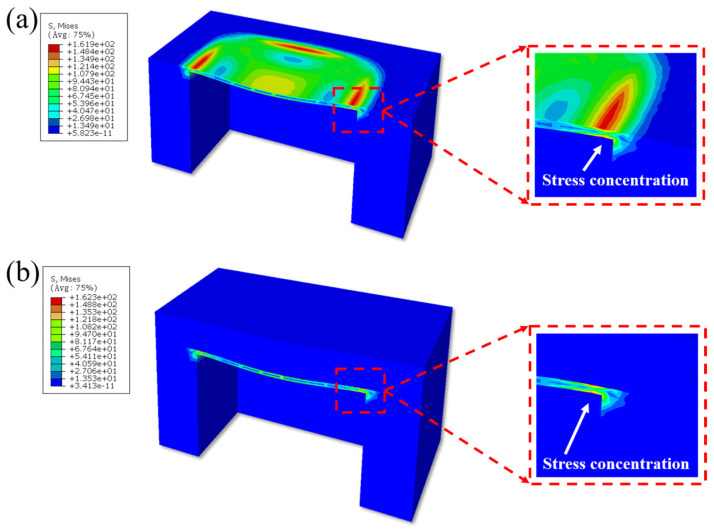
(**a**) Normal stress of the bare sensor; (**b**) normal stress of the Parylene/silicone oil composite sensor.

Figure 5a,b present the visualization results of the normal stress distribution on the sensor’s sensitive element before and after solid/liquid composite encapsulation. Prior to encapsulation, the maximum normal stress on the sensitive element was approximately 161.9 MPa, located at the midpoint of the four edges. Following encapsulation, the stress distribution pattern showed no significant change, with the maximum normal stress value being 162.3 MPa, representing a difference of only about 0.25%. This result demonstrates that the introduction of the solid/liquid composite encapsulation scheme does not alter the inherent, designed mechanical characteristics of the sensor chip. This stability in stress distribution is critically important, as it ensures that core performance parameters such as the sensor’s sensitivity and linearity do not drift due to packaging, which is essential for high-precision measurement applications.

**Figure 6:**
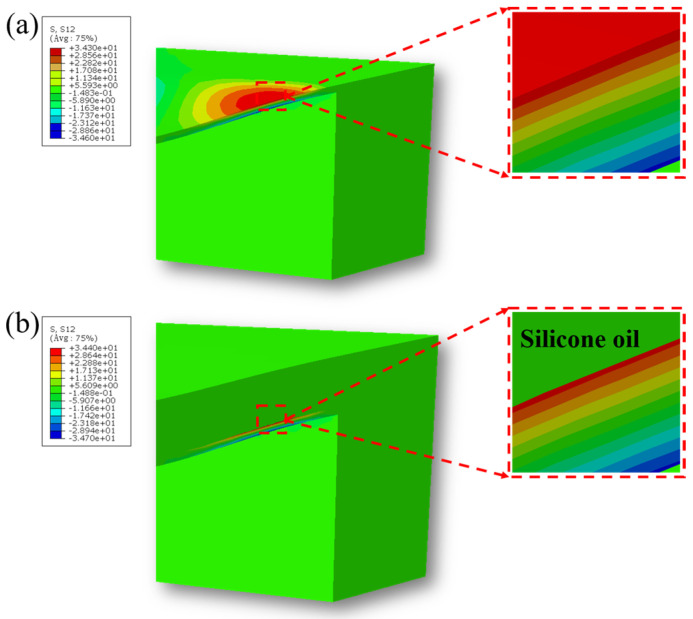
(**a**) Shear stress of the bare sensor; (**b**) shear stress of the Parylene/silicone oil composite sensor.

Furthermore, the shear stress distribution on the sensitive diaphragm before and after solid/liquid composite encapsulation is shown in Figure 6a,b. The results indicate that the introduction of the liquid silicone oil layer effectively blocks the transmission of in-plane shear stress between the solid encapsulation material and the sensor’s sensitive element. Consequently, the shear stress experienced by the sensitive diaphragm during pressure-induced deformation is nearly identical to that of the unpackaged state. This demonstrates that the liquid medium significantly mitigates the in-plane mechanical coupling between the encapsulation layer and the sensitive diaphragm at the solid/liquid interface. The significant reduction in shear stress transmission can further inhibit the risk of fatigue delamination at the encapsulation interface under cyclic pressure loading, thereby enhancing the long-term stability and reliability of the overall packaging structure.

Based on the Finite Element Method (FEM) simulation results, the solid/liquid composite encapsulation structure proposed in this study exhibits favorable characteristics. Firstly, the introduction of the liquid silicone oil layer in the encapsulation structure does not significantly increase the overall stiffness, demonstrating excellent flexibility, which successfully avoids the mechanical constraint of the sensor’s sensitive element. Secondly, external pressure is transmitted uniformly and effectively through the encapsulation layer to the sensitive element, ensuring consistent deformation during the pressure application process, thus guaranteeing the stable realization of the core sensing function. Further analysis of the deformation, normal stress, and shear stress distribution on the sensitive element reveals that the solid/liquid composite encapsulation demonstrates high mechanical compatibility. Specifically, the deformation mode and normal stress distribution of the sensitive element remain consistent before and after encapsulation, with changes in both deformation magnitude and maximum normal stress being less than 1%, indicating that the encapsulation does not cause significant interference with the sensitive element’s mechanical behavior. Crucially, the liquid silicone oil layer effectively isolates the shear forces between the encapsulation interface and the sensitive element, mitigating stress interference and the risk of delamination failure caused by shear coupling.

In summary, the simulation results demonstrate that the solid/liquid composite encapsulation exhibits an “invisible” mechanical characteristic, allowing the sensor to retain its original mechanical properties while simultaneously achieving the high reliability and stability required for long-term implantable applications.

### 3.2. Accuracy Testing

This study systematically compared the influence of single-layer Parylene encapsulation versus Parylene/silicone oil solid/liquid composite encapsulation on sensor performance by designing and fabricating a series of sensors with varying Parylene thicknesses. To determine the optimal encapsulation thickness parameter, five different Parylene film thicknesses were set: 0.5 μm, 1 μm, 1.8 μm, 3 μm, and 5 μm. All sensors were placed within the sealed pressure chamber of the testing system, where three-cycle performance tests were completed at an ambient.

**Figure 7:**
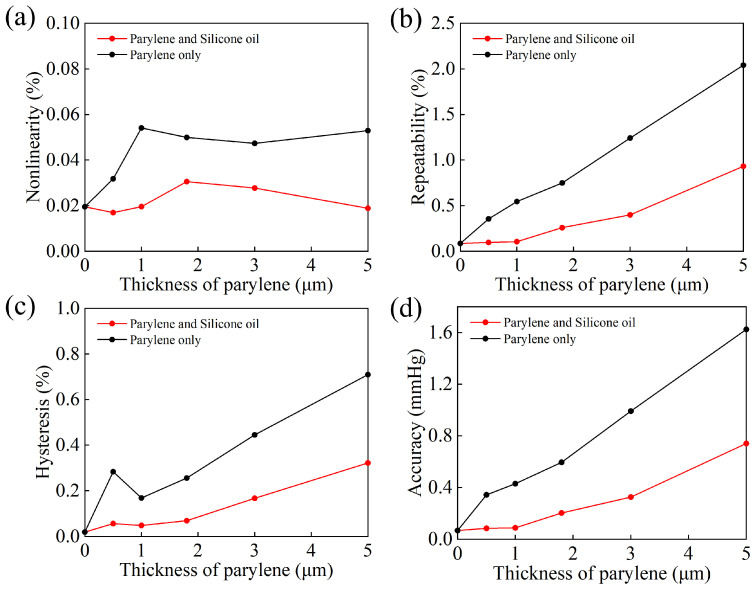
Error curves of unpacked sensors (0 μm), Parylene-only packed sensors, and Parylene/silicone oil packed sensors as a function of Parylene thickness: (**a**) nonlinearity error; (**b**) repeatability error; (**c**) hysteresis error; (**d**) accuracy.

Figure 7a–c systematically compare the variation in the sensor’s nonlinearity error, repeatability error, and hysteresis error with respect to Parylene thickness under three conditions: unpackaged (0 μm), single-layer Parylene encapsulation, and Parylene/silicone oil composite encapsulation. All three performance metrics exhibited highly consistent trends: as the Parylene thickness increased, the errors of the single Parylene-C encapsulated devices rose significantly. Notably, when the thickness exceeded 1 μm, the nonlinearity error surpassed 0.05%, and both repeatability and hysteresis errors showed clear amplification, indicating the detrimental effect of a thicker Parylene film on sensor performance. In contrast, the Parylene/silicone oil composite encapsulation effectively suppressed the accumulation of these errors with increasing thickness, maintaining low and stable performance across the entire test range. It is worth noting that in the thickness range below 1 μm, the three types of errors for the composite encapsulated sensors were virtually unaffected by the packaging, closely matching the unpackaged state. This further illustrates the significant buffering effect of the silicone oil layer in mitigating the additional stress and coupling effects introduced by the Parylene film. Figure 7d further calculates the relationship between the sensor’s overall accuracy (combined error) and the Parylene thickness. The results show that the accuracy of the single Parylene encapsulated devices deteriorates beyond 0.5 mmHg when the Parylene thickness exceeds approximately 1.8 μm. Conversely, the Parylene/silicone oil composite encapsulation is able to stably maintain an accuracy within 0.5 mmHg up to a thickness of 3 μm.

**Figure 8:**
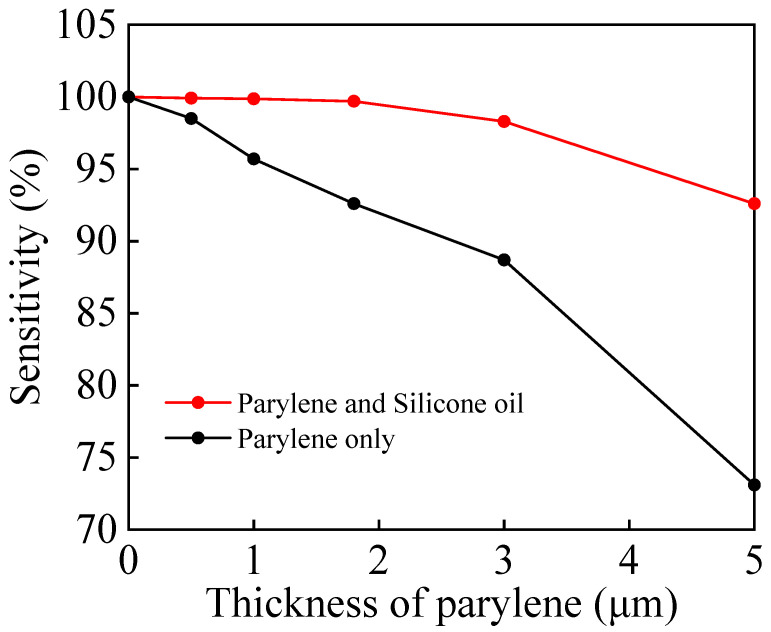
Sensitivity curves of unpacked sensors (0 μm), Parylene-only packed sensors, and Parylene/silicone oil packed sensors as a function of Parylene thickness.

Figure 8 illustrates the trend of decreasing sensor sensitivity with increasing Parylene film thickness under two encapsulation structures. Single-layer Parylene encapsulation exerts a greater impact on sensor sensitivity, causing it to drop sharply to approximately 73% when the film thickness reaches 5 μm. This is primarily due to the rigid solid Parylene film strongly inhibiting the deformation of the sensitive diaphragm. In contrast, the Parylene–silicone oil solid/liquid composite encapsulation effectively mitigates mechanical constraints on the Parylene film through the stress decoupling effect of the silicone oil layer. This results in a more gradual decline in sensitivity, maintaining above 92.5% even at a 5 μm thickness.

In summary, whether from the trends in the experimental data or the mechanical mechanisms revealed by simulations, the advantages of solid/liquid composite encapsulation extend beyond mere local optimization. It fundamentally alters the coupling mechanism between the encapsulation layer and the sensitive unit, transforming it from a “rigid solid-to-solid superposition” into a structure characterized by “flexible cushioning coupled with uniform load transfer”.

### 3.3. Stability Testing

For pressure sensors implanted in the human body, the packaging not only affects their initial accuracy and sensitivity but also directly impacts the device’s stability during long-term operation. While packaging provides essential mechanical protection and biological isolation, its material properties, interfacial stresses, and aging behavior over time can alter the sensor’s reference output. This causes signal offset—known as drift—even when the input pressure remains constant. Drift primarily encompasses zero-point drift and temperature drift, both representing the most prevalent and impactful stability issues for implantable devices. Given the temperature fluctuations within the body environment and the complexity of tissue fluid chemistry, the contribution of the encapsulation structure to drift cannot be overlooked. Therefore, to systematically evaluate the long-term stability characteristics of encapsulated sensors and clarify the impact of encapsulation on output consistency, this study conducted temperature drift and time drift tests on encapsulated sensors. To evaluate the improvement of long-term stability achieved by the encapsulation structure, comparative testing was performed using two device types: Parylene–silicone oil solid/liquid composite encapsulation and single-layer Parylene encapsulation. Additionally, to systematically investigate the effect of encapsulation thickness on stability, two sets of encapsulated devices with different Parylene film thicknesses were prepared for subsequent comprehensive analysis of barrier lifetime and drift characteristics.

**Figure 9:**
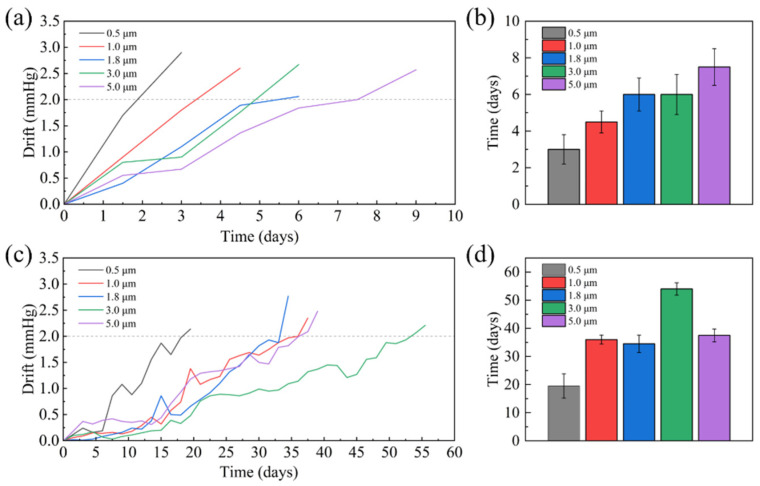
(**a**) Drift curve of Parylene packaged sensor with soaking time; (**b**) time to 2 mmHg drift for Parylene-only packaged sensor; (**c**) drift curve of Parylene/silicone oil packaged sensor with soaking time; (**d**) time to 2 mmHg drift for Parylene/silicone oil packaged sensor.

First, the encapsulated sensors were placed in a constant-temperature saline solution at 37 °C to simulate the in vivo environment, and anti-permeation lifespan and zero drift tests were conducted. According to the accuracy standards for implantable pressure sensors, a device is considered failed when its error exceeds 2 mmHg. Figure 9a–d show the drift curves of the Parylene-only packed sensors and the Parylene/silicone oil packed sensors with soaking time. The results indicate that as the Parylene film thickness increases, the anti-permeation lifespan improves for both encapsulation types. For the Parylene-only packed sensors, when the film thickness is 5 μm, the anti-permeation lifespan reaches 7 days. For the Parylene/silicone oil packed sensors, when the Parylene film thickness exceeds 0.5 μm, the anti-permeation lifespan can exceed 30 days.

The temperature drift test of the sensor was conducted using a constant-temperature water bath system within the human body temperature range of 34 °C to 42 °C. During the test, the sensor was immersed in physiological saline, and its output changes were recorded at temperature points increasing in increments of 2 °C to evaluate the influence of encapsulation on the sensor’s temperature stability.

**Figure 10:**
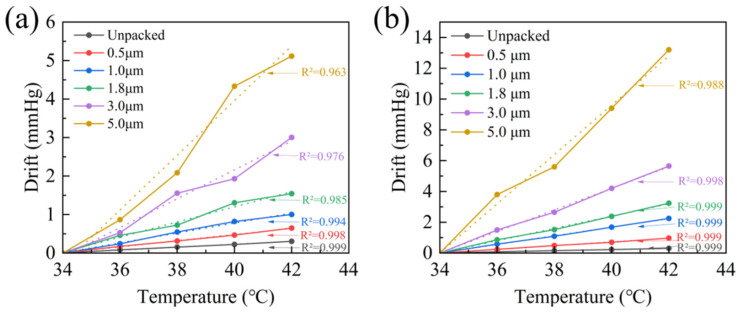
(**a**) Output drift curve of Parylene-only packed sensors with temperature; (**b**) output drift curve of Parylene/silicone oil packed sensors with temperature.

Figure 10a,b show the temperature drift curves of the Parylene-only packed sensors and the Parylene–silicone oil packed sensors, respectively. Within the temperature range of 34 °C to 42 °C, the drift magnitude of the sensors is positively correlated with the Parylene film thickness—that is, a thicker film results in greater sensor drift. Under the same Parylene film thickness condition, the temperature drift magnitude of the Parylene–silicone oil composite encapsulated sensors is approximately half that of the Parylene-coated sensors. The temperature drift curves of the composite encapsulated sensors exhibit significantly superior linear fitting (with higher R^2^ values), a characteristic that is more conducive to the accurate implementation of subsequent temperature compensation.

The Parylene/silicone oil composite encapsulation demonstrates significant comprehensive advantages in implantable pressure sensor applications. In zero-point drift tests conducted in a simulated saline environment at 37 °C, the thickness of the Parylene film determines the baseline anti-permeation duration, while the introduction of silicone oil substantially extends the sensor’s effective anti-permeation lifespan from 7 days (with Parylene encapsulation alone) to over 30 days by providing additional hydrophobic barriers and defect-repair effects, thereby meeting the requirements for long-term implantation. Furthermore, drift characteristic analysis within the 34 °C–42 °C temperature range reveals that although drift magnitude increases with Parylene film thickness, the temperature drift curves of the composite encapsulation exhibit excellent linear fitting (R^2^ value close to 0.999). This high linearity is likely attributed to the homogenizing effect of the silicone oil layer on thermal stress distribution, which ensures a stable linear relationship between thermal drift and temperature. This provides a solid foundation for subsequent high-precision temperature compensation, ultimately confirming the dual effectiveness of the composite encapsulation strategy in enhancing both the long-term stability and measurement accuracy of the sensor.

## 4. Conclusions

This study proposes a Parylene/silicone oil solid/liquid composite encapsulation technique for implantable pressure sensors. Repeated testing confirmed that the solid/liquid composite encapsulation remained intact under pressure, with no displacement of the silicone oil layer. This structural integrity demonstrates that the device effectively meets the requirements for in vivo pressure monitoring. FEA simulations and experimental results confirm that the liquid silicone oil layer effectively decouples interface shear forces, mitigating stress interference and delamination risks. Compared to single-layer Parylene, the composite structure suppresses the accumulation of nonlinear, repeatability, and hysteresis errors, maintaining high accuracy (within 0.5 mmHg) even at a 3 μm Parylene thickness. Additionally, the hydrophobic and defect-filling properties of silicone oil extended the sensor’s functional lifespan in 37 °C saline from 7 days to over 30 days. The high linearity of temperature drift (R^2^ ≈ 0.999) further facilitates precision compensation. This approach provides a robust and high-performance solution for the long-term encapsulation of implantable biomedical devices.

## Data Availability

The original contributions presented in this study are included in the article. Further inquiries can be directed to the corresponding authors.
